# Tailor-Made Ezrin Actin Binding Domain to Probe Its Interaction with Actin *In-Vitro*

**DOI:** 10.1371/journal.pone.0123428

**Published:** 2015-04-10

**Authors:** Rohini Shrivastava, Darius Köster, Sheetal Kalme, Satyajit Mayor, Muniasamy Neerathilingam

**Affiliations:** 1 Protein Technology Core, Centre for Cellular and Molecular Platforms NCBS-TIFR, GKVK Post, Bangalore, India; 2 NationalCentre for Biological Sciences Tata Institute of Fundamental Research GKVK, Bangalore, India; University of South Florida College of Medicine, UNITED STATES

## Abstract

Ezrin, a member of the ERM (Ezrin/Radixin/Moesin) protein family, is an Actin-plasma membrane linker protein mediating cellular integrity and function. *In-vivo* study of such interactions is a complex task due to the presence of a large number of endogenous binding partners for both Ezrin and Actin. Further, C-terminal actin binding capacity of the full length Ezrin is naturally shielded by its N-terminal, and only rendered active in the presence of Phosphatidylinositol bisphosphate (PIP2) or phosphorylation at the C-terminal threonine. Here, we demonstrate a strategy for the design, expression and purification of constructs, combining the Ezrin C-terminal actin binding domain, with functional elements such as fusion tags and fluorescence tags to facilitate purification and fluorescence microscopy based studies. For the first time, internal His tag was employed for purification of Ezrin actin binding domain based on *in-silico* modeling. The functionality (Ezrin-actin interaction) of these constructs was successfully demonstrated by using Total Internal Reflection Fluorescence Microscopy. This design can be extended to other members of the ERM family as well.

## Introduction

Cytoskeleton proteins are involved in several cellular processes such as maintaining cell shape, motility or cell division and provide structural support to the plasma membrane [1, 2]. Actin, one such cytoskeleton protein, is highly conserved and abundantly available in eukaryotic cells [3]. Particularly, cortical actin, which underlies the inner surface of the plasma membrane, is important for various membrane related processes like endocytosis, migration and signal transduction from the surface to the interior of the cell. Cortical actin is connected to the plasma membrane through linker proteins such as, Ezrin, Radixin and Moesin (ERM). The common structure of these linker proteins comprise a) an N-terminus membrane binding FERM domain {band 4.1 protein(F), Ezrin(E), Radixin(R) and Moesin(M)}, b) an intermediary domain and c) a C-terminus F-Actin binding domain [4]. Here, the FERM domain binds to the plasma membrane and the C-terminal F-actin binding domain interacts with actin to form ternary complexes, which are involved in signal transduction. However, studying these interactions *in-vivo* is difficult due to situational association of ERM proteins like Ezrin to multiple proteins in the cell [1]. To overcome this, interactions between plasma membrane-Ezrin-actin ternary complexes are analyzed *in*-*vitro* using supported lipid bilayers (SLB) [5, 6]. One of the primary advantages of using SLBs is the two-dimensional platform of this model membrane system that readily allows protein-membrane interactions to be imaged and characterized with a small amount of protein [7, 8].

**Fig 1:**
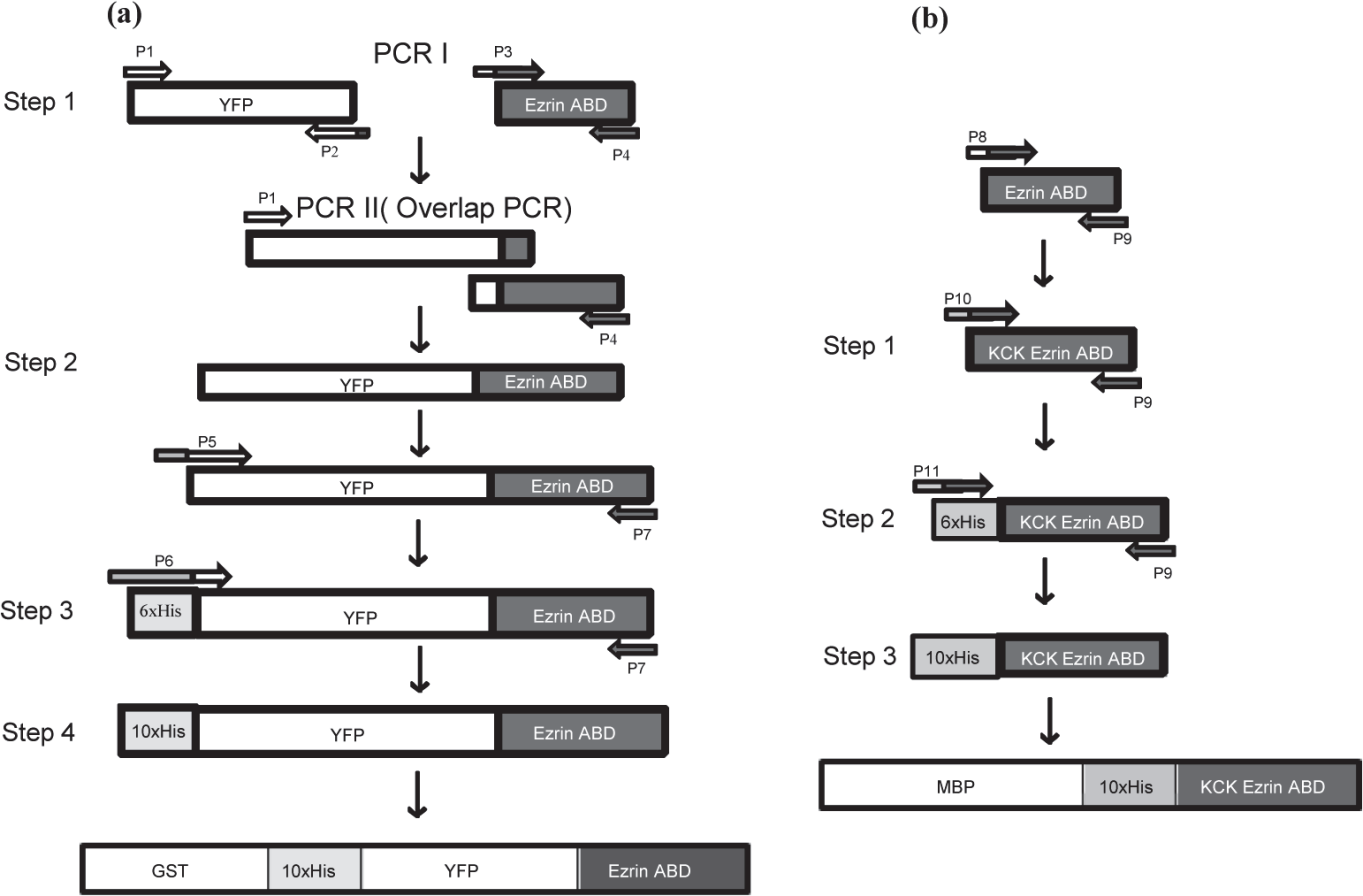
Schematic representation for generation of bright ezrinABD and dark ezrnABD. (a) Step 1. Amplification of templates for splicing overlap PCR, Step 2. Product of splicing overlap PCR (S3 Fig YFP-ezrinABD), Step 3. Addition of 6xHis at the N-terminal of YFP-ezrinABD (S4A Fig), Step 4. Addition of 10xHis at the N-terminal of YFP-ezrinABD (S4B Fig), (b) Step 1. Amplification of KCK-ezrinABD, Step 2. Addition of 6xHis at the N-terminal of KCK-ezrinABD (S4C Fig), Step 3. Addition of 10xHis at the N-terminal of KCK-ezrinABD (S4D Fig).

Full length Ezrin is normally in a dormant confirmation masking the C-terminal actin binding domain with its own N-terminal FERM domain [9, 10] resulting in the absence of interaction with Actin [11]. Conformational change in full length Ezrin occurs after addition of Phosphatidylinositol bisphosphate (PIP2) [4, 5] which binds to the N terminal of the full length Ezrin and thus promotes binding to actin. In an alternative approach, we chose to employ the Ezrin C-terminal domain alone in combination with a lipid anchor 10xHis and a fluorescent tag in order to facilitate *in vitro* studies with Total Internal Reflection Fluorescence (TIRF) microscopy. In particular, we present a design comprising a combination of affinity tags that allow high level expression and affinity purification of the Ezrin actin binding domain (ABD) without losing its functionality. We tested two constructs, viz., 1) 10xHis-Yellow Fluorescent Protein (YFP)-ezrinABD with Glutathione S-transferase (GST) and 2) 10xHis-Lysine-Cystine-Lysine (KCK)-ezrinABD with Maltose Binding Protein (MBP) for expression, solubility, purity and subsequent functionality test by TIRF microscopy (S1 and S2 Figs). N-terminus membrane binding FERM domain was replaced by 10xHis in above mentioned constructs. While construct1 was fluorescently labeled with YFP and called bright ezrinABD, construct 2 has a non-fluorescent KCK sequence instead, which we refer to as dark ezrinABD (Fig 1). By demonstrating this successfully, we show how a strategic combination of affinity tags can be instrumental for large scale purification of complex, multifunctional protein constructs without addition of PIP2 or phosphorylation.

## Materials and Methods

### Generation of constructs with fusion tags

YFP was amplified using forward primer YFP I, reverse primer YFP I, and spliced with Ezrin, which was amplified using forward primer Ezrin I, reverse primer Ezrin I (S1 Table). The resulting PCR product (YFP-ezrinABD) (S3 Fig) was gel purified and digested with *NdeI* and *BamHI*. This insert was cloned into pET-15b vector containing 6xHis at the N-terminal, using *NdeI* and *BamHI* sites. 6xHis-YFP-ezrinABD was confirmed by restriction digestion and sequencing. To create constructs bearing 10xHis tag, YFP-ezrinABD with 6xHis was amplified with forward and reverse primers containing additional 4xHis residues. The resulting product (bright ezrinABD) was purified and ligated into pGEX-4T-1 (Fig 1A) and pMAL-c5X. Clones were confirmed by restriction digestion and sequencing. Similarly, KCK-ezrinABD (dark ezrinABD) construct was PCR amplified, 10xHis was added at N-terminal and cloned into vector pMAL-c5X (Fig 1B) and pGEX-4T-1.

### Expression and purification of recombinant constructs

*E coli* BL21 (DE3) transformants containing GST tagged bright ezrinABD were grown in 2xYT media containing ampicillin (100 μg/ml) to an OD_600_ of 0.4–0.6 at 37°C. Further, cultures were induced with 0.3 mM IPTG and grown at 18°C overnight. Post-induction, the cells were harvested by centrifugation and resuspended in 50 mM NaH_2_PO_4_,150 mM NaCl,10 mM imidazole,1 mM β-Mercaptoethanol pH 7.5 and lysed by sonication (15 seconds on, 30 second off; 10 Cycles). This was followed by centrifugation at 13,000 × *g* for 20 min at 4°C twice. The supernatant was loaded onto a Ni^2+^-nitrilotriacetic acid column(Ni-NTA) (GE healthcare), which was washed with a gradient of 50 mM NaH_2_PO_4_, 150 mM NaCl, 10 mM imidazole, 1mM β-Mercaptoethanol and 50 mM NaH_2_PO_4_, 300 mM NaCl, 50 mM imidazole, 1 mM β-Mercaptoethanol, pH 7.5 spanning 20 Column Volume (20 CV). The GST tagged bright ezrinABD protein was eluted using linear gradient of buffer containing 50 mM NaH_2_PO_4_, 300 mM NaCl,10 mM imidazole, 1 mM β-Mercaptoethanol and 50 mM NaH_2_PO_4_, 150 mM NaCl, 500 mM imidazole, 1 mM β-Mercaptoethanol pH 7.5 spanning 10 CV. Further, the protein was concentrated and buffer exchanged using 30 kDa Amicon (Millipore) and quantified using Nanovue plus (GE healthcare). GST tag was cleaved using 10 U/mg of thrombin for 8–10hr at 4°C.

Similarly, MBP-tagged dark ezrinABD was transformed into *E*. *coli* BL21 (DE3) star strain and expression was induced using 0.4 mM IPTG at 30°C. Post induction, the harvested cells were resuspended in 50 mM NaH_2_PO_4_, 150 mM NaCl, 10 mM imidazole, and 1 mM β-mercaptoethanol pH 7.5 and sonicated. Further, purification protocol was performed similar to GST tagged bright ezrinABD and MBP-tag was cleaved using Factor Xa.

### Size exclusion chromatography (SEC) analysis

Bright and dark ezrinABD were fractionated on a Superdex 75 10/300 GL column (GE Healthcare) equilibrated with 50 mM Tris-Cl, 300 mM NaCl, 1 mM DTT. The elution was carried out at a flow rate of 1 ml/min and the absorbance at 280 nm was continuously monitored using AKTA purifier (GE). Standard molecular weight marker was used to validate the column.

### Purification of GST tagged and MBP-tagged proteins

GST tagged bright ezrinABD was purified using GSTrap HP prepacked column (5 ml), which was equilibrated with 50 mM NaH_2_PO_4_, 150 mM NaCl, 1 mM DTT pH 7.4 and washed in buffer containing 50 mM NaH_2_PO_4_, 300 mM NaCl, 1 mM DTT pH 7.4. The elution of GST tagged bright ezrinABD was done with 50 mM Tris-Cl, 10mM Glutathione, and 1 mM DTT pH 7.4.

For MBP-tagged dark ezrinABD, dextrin sepharose beads (2 ml) were used. The beads were equilibrated with equilibration buffer (20 mM Tris-Cl, 200 mM NaCl, 1 mM EDTA and 1mM DTT pH 7.4) and the lysate was loaded on to the column. This was followed by washing with equilibration buffer and eluted with 10 mM Maltose.

### Supported lipid bilayers formation

Supported lipid bilayers (SLBs) were formed by fusion of Small Unilamelar Vesicles (SUV) with a lipid composition of 98%PC (phosphatidylcholine (99%) (Egg, Chicken), Avanti Polar Lipids, Alabaster, AL, USA) and 2% DGS-Ni-NTA (18:1 DGS-NTA (Ni^2+^ 1, 2-dioleoyl-sn-glycero-3-(N-(5-amino-1-carboxypentyl) iminodiacetic acid) succinyl) (nickel salt), Avanti Polar Lipids, Alabaster, AL, USA) on freshly cleaned silica glass slides (Lab-Tek II#1.5 chambered coverglass, 8 well (Thermo Fisher Scientific, USA)). First, lipids were mixed in chloroform, dried with nitrogen gas followed by 3 hours in a vacuum desiccator, and re-hydrated in SUV buffer (20 mM HEPES, 150 mM NaCl, 5%sucrose, pH 7.2) to a final lipid concentration of 4 mM. After the lipid mix underwent 10 cycles of freeze-thaw, SUVs were formed by the extrusion method using a membrane with 80 nm diameter holes (Mini-Extruder, Avanti Polar Lipids, Alabaster, AL, USA). Glass slides were immersed in 2% Hellmanex III solution for 30 min at 50°C and rinsed thoroughly in MilliQ water. Further, it was immersed in 3M NaOH for 15 min, rinsed thoroughly in MilliQ water and blow dried with nitrogen gas. For SLB formation, 15μl SUV mix was added to 100 μl SLB formation buffer (150 mM NaCl, 20 mM HEPES, pH 5.5) in each well and incubated for 20 min followed by thorough washing with SLB buffer (20 mM HEPES, 150 mM NaCl, pH 7.2). For F-actin binding experiments, SLB buffer was exchanged with KMEH (50 mM KCl, 1 mM MgCl_2_, 1 mM EGTA, 20 mM HEPES, pH 7.2).

### Actin polymerization

Actin was purified from chicken breast following the protocol from Spudich and Watt [12] and labeled with NHS-Cy3 as described by Kellogg et.al [13]. The chicken breast was purchased in a chicken shop close to our laboratory (Venky Chicken Center, D Rajgopal Road, Kodigehali, Bangalore, India (geographic coordinates: 13.061640 latitude, 77.574763 longitude)) which freshly and professionally butchers chicken originating from a local farmer, and was directly used for actin and myosin extraction. 10 μM G-actin (10% Cy3 labeled) was polymerized in 20 μl of polymerization buffer (50 mM KCl, 1 mM MgCl_2_, 1 mM EGTA, 20 mM β-mercapto-ethanol, 1 mg/ml BSA, 1 mM ATP, 20 mM HEPES, pH 7.2) for 30 min at RT, then added to the SLB containing well filled with 100 μl KMEH. Final F-actin concentration on the SLB was 400 nM. 0.1–1.2 nM of bright ezrinABD or dark ezrinABD was added to the SLB, and binding of F-actin was checked with TIRF microscopy.

### Imaging and data analysis

All images were acquired using TIRF microscopy on Nikon Eclipse Ti equipped with a motorized TIRF adapter (Nikon), fed by a fiber coupled monolithic laser combiner with four lasers at 405, 488, 561 and 631 nm (MLC 400, Agilent Technologies Inc, Santa-Clara, CA, USA). All measurements were made at 20°C. Images were collected at a fixed magnification using 100 × 1.49NA objective lens, yielding a pixel size corresponding to 154 nm. F-actin and bright ezrinABD images were acquired with 200 ms exposure time at minimal laser intensities to reduce photo bleaching.

To estimate the ratio of F-actin bound to SLBs, we analyzed the actin images with a self-developed code in Matlab (MathWorks, Natick, MA, USA). Briefly, the laplacian (divergence of the gradient) of the actin intensity was computed and subjected to a threshold selecting for high negative divergence (intensity 'sinks') and isolated pixels were discarded as noise. This procedure allowed us to reliably segment pixels of the image occupied by actin filaments. F-actin occupation was then computed as the area fraction of these pixels.

### In-silico studies

The structure prediction of the constructs was performed using the HMM (Hidden Markov Model) based software T-SAM 08. The structures with the highest E values were selected and were visualized using PyMOL.

## Results

### Large scale expression and purification of GST-tagged bright ezrinABD and MBP-tagged dark ezrinABD

Constructs of YFP ezrinABD and KCK ezrinABD were cloned in pET-15b. These constructs were expressed in different strains of *E*.*coli* as listed in S3 Table. However, no yield was observed with pET-15b during the initial optimization with different IPTG concentrations and various temperatures. Bright and dark ezrinABD were cloned in both pGEX-4T-1 (containing GST) and pMAL-c5X (containing MBP) to check the influence of GST and MBP fusion tags on solubility and to improve expression. Expression of bright ezrinABD in pGEX-4T-1 resulted in soluble protein and relatively better yield than pMAL-c5X in BL21 (DE3). Although dark ezrinABD in pGEX-4T-1 expressed in *E*. *coli* BL21 (DE3) gave higher yield, it was found to be insoluble. In contrast, dark ezrinABD cloned in pMAL-c5X gave soluble protein when expressed in *E*. *coli* BL21 (DE3) star whereas yield was lesser than that of pGEX-4T-1.

**Fig 2:**
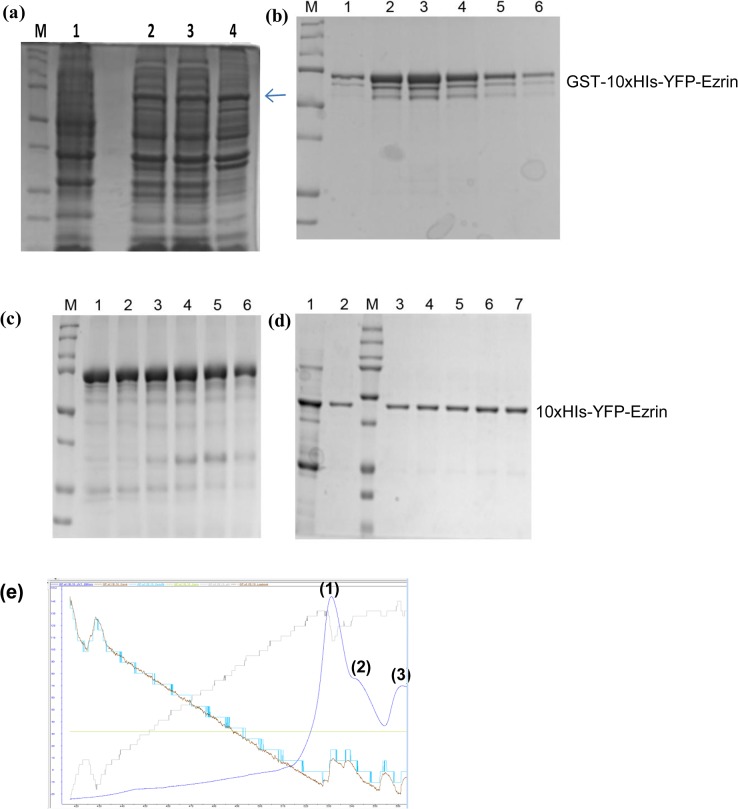
SDS-PAGE analysis of GST tagged bright ezrinABD. (a)12% SDS-PAGE gel showing the expression of 69 kDa protein, lane M; Protein marker, lane 1; Uninduced, lane 2; Total, lane 3; Supernatant (soluble), lane 4; Flow through, (b) GST-tagged bright ezrinABD(69 kDa) purified using GSTrap HP column, lane M; Protein marker, lane 1–6; eluted fractions, (c) GST-tagged bright ezrin purified using His Trap HP column, lane M; Protein marker, lane 1–6; eluted fractions, (d) Gel permeation of 42 kDa bright ezrinABD, lane M; Protein marker, lane 1; thrombin cleaved GST-tagged bright ezrinABD, lane 2–7; purified fractions, (e) AKTA chromatogram of Gel Permeation of bright ezrinABD after cleavage of GST-tag Peak (1) GST-tagged bright ezrinABD, Peak (2) bright ezrinABD, Peak (3) GST.

**Fig 3:**
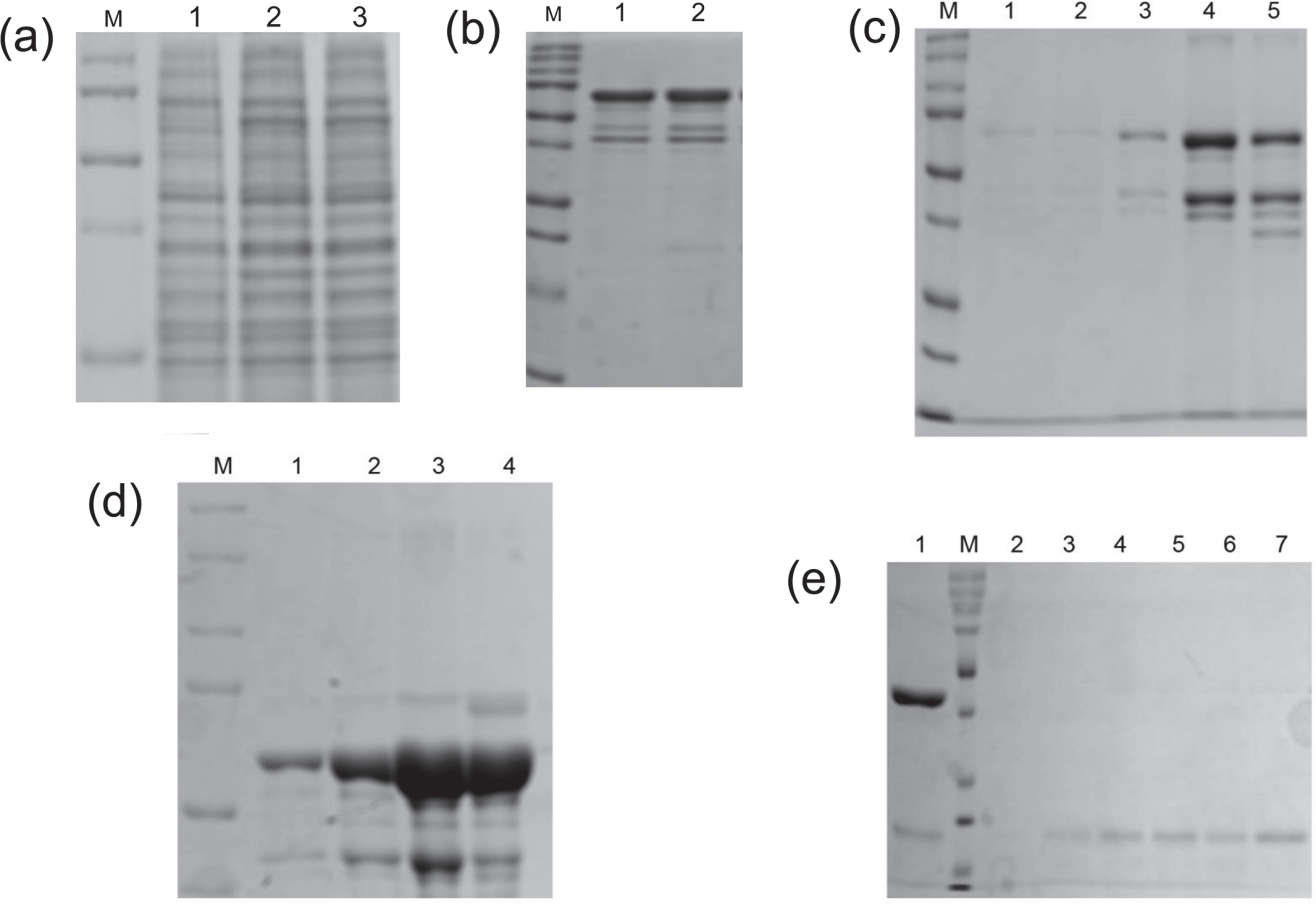
SDS-PAGE analysis of MBP-tagged dark ezrinABD. (a)12% SDS-PAGE gel showing the expression of 57 kDa protein, lane M; Protein marker, lane 1; Uninduced, lane 2; load, lane 3; Flowthrough, (b) MBP-tagged dark ezrinABD purified using Dextrin Sapharose,lane M; Protein marker, lane 1&2; Washthrough, (c) lane M; Protein marker, lane 1–5; eluted fractions, (d) MBP-tagged dark ezrinABD purified using His Trap HP column, lane M; Protein marker, lane 1–4; eluted fractions, (e) The purification of 15 kDa dark ezrinABD protein using Dextrin Sepharose, lane M; Protein marker, lane 1; Load, lane 2–7; eluted fractions.

The optimum temperature and IPTG concentration for expression of GST tagged bright ezrinABD (10xHis-YFP-ezrinABD-construct1) and MBP-tagged dark ezrinABD (10xHis-KCK-ezrinABD-construct2) were different. For expression of GST tagged bright ezrinABD in *E*. *coli* BL21 (DE3), 0.3 mM IPTG concentration at 18°C was employed, (Fig 2(A)). Whereas 0.4 mM IPTG concentration at 30°C was optimum for expression of MBP-tagged dark ezrinABD in *E*. *coli* BL 21(DE3) star (Fig 3(A)).

**Fig 4:**
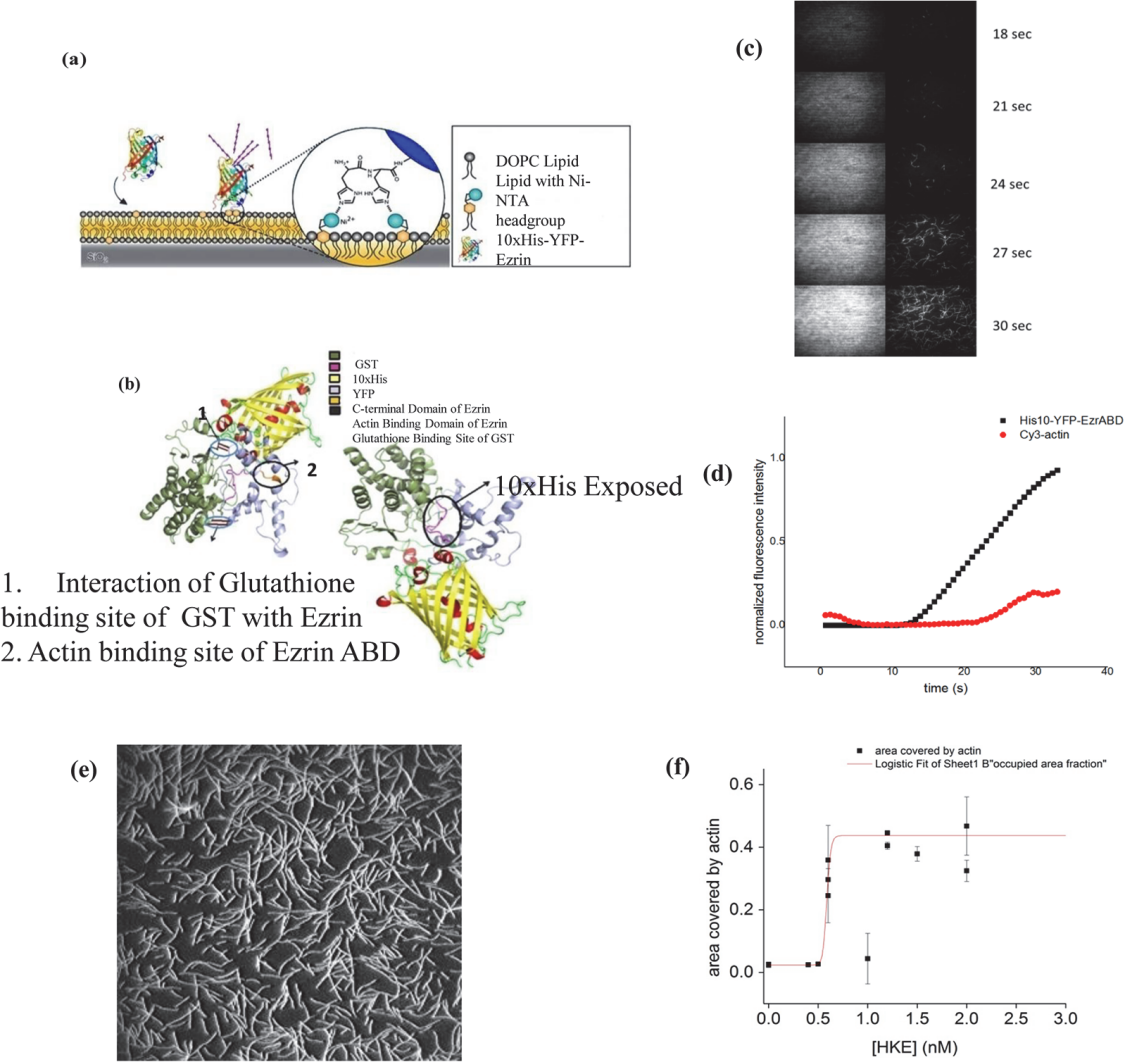
GST. **(a-d) ezrinABD interaction**: (a) binding of bright ezrinABD to SLB, (b) Predicted structure of GST-tagged bright ezrinABD, (c) images from TIRF microscopy, (d) graphical presentation of cy3 Actin binding to Ezrin in different time frame; **(e, f) Saturation of ezrinABD and actin binding**: (e) TIRF microscopy image of f-actin binding to SLB via HKE (dark ezrinABD). (f) Plot of actin binding (area fraction covered by actin) at different concentrations of HKE; red line is a fit of a logistic function, to visualize the sharp onset of actin binding to the SLB at C_HKE_ > 0.7 nM, data points represent mean and standard deviation of individual experiments.

We purified GST tagged bright ezrinABD and MBP-tagged dark ezrinABD using Glutathione 4B and dextrin sepharose beads, respectively. However, purification of GST tagged bright ezrinABD using Glutathione 4B beads rendered low yield (0.25 mg/ml) after purification (Fig 2(B)). Therefore, the non-binding of GST tagged bright ezrinABD to GST beads was further analysed using *in-silico* studies. The structure of the protein was predicted and the model with the best E-value was chosen for analysis. As indicated in Fig 4B, it was observed that the GST binding site (residues 33–37) was masked by the residues in both ezrinABD domain (residues 480–600) and YFP domain (residues 237–479). The residue E34 was found to be directly interacting with K315 of YFP. The residues E37 and R35 were indirectly interacting with Q316 of YFP and R551 of ezrinABD domain. This indicated that GST tag cannot be used for purification. On the other hand, the flexible internal 10xHis-tag was observed to be exposed to the solvent. As this exposed 10xHis-tag could bind to Ni^2+-^NTA matrices, GST tagged bright ezrinABD was purified using Histidine affinity chromatography (Fig 2(C)). Further, the purified protein was then subjected to thrombin cleavage with 10 U/mg of thrombin for 8–10 hr at 4°C and purified using size exclusion chromatography (Fig 2(D)). GST tag and cleaved 10xHis-YFP-Ezrin (bright ezrinABD) was separated by size exclusion chromatography as shown in Fig 2(E). Bright ezrinABD was purified as monomer which indicated no intramolecular interaction. The concentration of purified bright ezrinABD was 1 mg/ml after quantification with Nano plus (GE healthcare). Purification of MBP-tagged dark ezrinABD using dextrin sepharose beads showed loss of protein during wash, suggesting very less binding of MBP-tag to the sepharose. As the total protein concentration in the load (10ml) was about1mg/ml, while wash through showed concentration of 0.4mg/ml for the concentrated sample (5ml was concentrated to 1ml) and 0.2mg/ml for the eluted sample, yielding 0.1 mg/ml of dark ezrinABD after purification (Fig 3(B and 3C)). Similar to YFP-Ezrin, 10xHis internal tag was used to purify MBP-tagged dark ezrinABD (Fig 3(D)). Further, factor Xa was used to cleave MBP-tag from dark ezrinABD with 6.25 U/mg for 8 hr at 4°C and purified using gel permeation. After gel permeation MBP was not completely separated from dark ezrinABD, consequently MBP was trapped by dextrin sepharose beads and final concentration of dark ezrinABD after purification was 1 mg/ml (Fig 3(E)). After cleavage of MBP from dark ezrinABD, interaction of free MBP with dextrin sepharose beads confirmed masking of MBP binding site in MBP-dark ezrinABD.

### Confirmation of Ezrin functionality by TIRF

The functionality of both the ezrinABD constructs was confirmed by an actin binding assay. Here, Cy3 labeled F-actin was allowed to settle on SLBs doped with 2 mol% DGS-NiNTA lipids at different concentrations of the ezrinABD constructs, and the amount of membrane bound F-actin was estimated from the images obtained by TIRF microscopy (see Materials and Methods section). In the absence of bright and dark ezrinABD, no F-Actin signal was observed in the TIRF field indicating that F-Actin does not bind to bare SLBs. In contrast, after addition and binding of bright and dark ezrinABD to SLBs, F-actin was rapidly detected in the TIRF field indicating ezrinABD mediated binding of F-actin to SLBs (Fig 4). This confirms the purity and functionality of bright and dark ezrinABD.

## Discussion

Ezrin has three domains, out of which, two domains participate in signal transduction namely N-terminal FERM domain and C-terminal actin binding domain. Dormant state of Ezrin involves masking of C-terminal actin binding domain by its N-terminal FERM domain, which renders inability to bind with actin. Furthermore, Ezrin interacts with other endogenous proteins *in-vivo*, thus making analysis difficult [1] i.e. N-terminal and C-terminal are engage in interactions with membrane proteins and F-actin, as well as intramolecular and intermolecular interactions. Ezrin gets activated by the binding of PIP2 or by phosphorylation at the C-terminal threonine. When Ezrin is activated, it binds directly to the cortical actin cytoskeleton. If there is dephosphorylation of the C-terminal threonine and reduction in PIP2levels, it leads to inactivation of ERM and their release from the cell membrane [14].

To reproduce plasma membrane-Ezrin-actin interactions *in-vitro*, a system that consists of a minimal set of defined components is used. The preparation of cytoskeleton-membrane linker proteins like Ezrin for *in-vitro* studies is challenging with respect to protein solubility and high yield. For *in-vitro* study, use of microtiter plate and surface plasmon resonance technology (SPR) encountered less binding and lower solubility of recombinant Ezrin protein [15]. In our study, various fusion tags GST, MBP and 10xHis were employed to enhance the expression and solubility as shown in S2 Fig.

The supported lipid bilayer (SLB) is an experimental research design particularly useful for studies associated with plasma membrane and its related proteins *in vitro*. SLB indicates structural and dynamic properties like cell membrane but former is better with respect to this study as one can perform restricted set of experiments without involvement of accessory components of native membranes, still proving functionality comparable to latter. SLB can overcome the problems that come into picture because of surface sensitivity of cell membranes during microscopy and surface sensitive spectroscopies [16]. Due to less understanding of suitable binding partners between recombinant proteins and SLB these studies are restricted, but Nickel-Chelating lipids have been observed to bind efficiently with fusion proteins containing histidine tag [17]. These artificial chelating lipid like nickel also reduce the trouble in fixing the biological samples. We facilitate this study by using 10xHis in both bright and dark Ezrin.

To observe the interaction between plasma membrane-Ezrin-Actin ternary complexes with fluorescence microscopy, modifications like Yellow fluorescent protein (YFP) and Lysine-Cystine-Lysine (KCK) were used in an arrangement shown in S1 Fig. KCK serves as a dark spacer, whose cysteine can be used to label the protein with maleimide functionalized fluorophores. Initially, as only the ezrinABD domain of Ezrin was employed for expression, it posed to be a difficult target [18] and expression trials did not result in soluble protein (Data not shown). Previous studies suggest GST as a favorable fusion tag for Ezrin and maltose binding protein (MBP) for solubilizing the protein and enhancing expression level [19, 20]. These tags were thus incorporated in bright ezrinABD and dark ezrinABD respectively. Because of the intramolecular interaction of full length Ezrin rendering dormant confirmation, only the ezrinABD domain was employed. Further, N-terminal domain of Ezrin was replaced by 10xHis for binding to Ni-NTA lipids containing supported lipid bilayers (SLBs) (Fig 4(A)). This approach allows us to study the effect of the ezrinABD in an isolated manner. The use of a 10xHis anchor was shown to mediate a very stable protein-lipid binding which stays for longer period of time [16]. When the constructs were expressed using pGEX-4T-1 and pMAL-c5X (with bright ezrinABD and dark ezrinABD) in *E*. *coli* BL21 (DE3) and *E*. *coli* BL21 (DE3) star, respectively, it was observed that GST tagged dark ezrinABD was insoluble and expression levels of GST tagged bright ezrinABD and dark ezrinABD were high as compared to their MBP-tagged variants. At lower IPTG concentration and temperature conditions, GST tagged bright ezrinABD expression was enhanced along with its solubility (Fig 2A). GST tagged bright ezrinABD was purified using affinity chromatography during which longer incubation time was required for binding to the glutathione sepharose column. Although GST tagged control bound to the glutathione 4B beads with high affinity, GST tagged bright ezrinABD showed weak interaction (Fig 2B). As expected, the supernatant and flow through contained yellowish green fluorescence indicating properly folded glutathione binding site and YFP, and hence a properly folded protein. However, as the binding to glutathione beads was not successful, GST could not be used for purification of the bright ezrinABD. ERM has the property of dimers or oligomers formation with which, Ezrin can form heterotypic associations [15]. Our findings indicated that the non-binding of GST could be due to masking of glutathione binding site by the YFP ezrinABD region of the construct by intramolecular interaction. This was predicted by the homology modeling, where the interaction between ezrinABD and GST was elucidated (Fig 4B). In the same model, we observed that 10xHis tag is a random coil and suspected that it could be exploited for purification. Indeed, GST tagged bright ezrinABD internal 10xHis was used to purify the protein successfully by affinity purification using affinity (Ni^+2^column) and size exclusion chromatography (Fig 2C). The GST tag ensured the solubility, and was cleaved later with thrombin to get 10xHis-YFP-ezrinABD (bright ezrinABD). Using the internal 10xHis we also purified MBP-tagged dark ezrinABD along with MBP because of improper binding of MBP-tag with dextrin sepharose beads. MBP-tag has not been used for ezrinABD purification till now. However, in the present study MBP helped for solubility but it failed in affinity purification.

The SDS-PAGE analysis of His-affinity purified protein revealed YFP-ezrinABD, a protein of 69 kDa, to run at 75 kDa along with other impurities. A similar shift in gel mobility of GST tagged Ezrin was observed by Algrain *et al*. as well as Gary and Bretschert during purification of GST tagged Ezrin, which was suggested due to the presence of acidic amino acid [10, 21]. As impurities were observed after affinity purification of YFP-ezrinABD, ion exchange chromatography was performed, however, this resulted in less yield and the impurities still persisted. It has been observed that Ezrin is susceptible for proteolysis [18] which correlates to our observation during purification of GST and MBP-tagged YFP-ezrinABD and KCK-ezrinABD respectively (data not shown).

To study the functionality of bright ezrinABD the protein was bound to SLB (doped with 2 mol% DGS-Ni-NTA), and then Cy3 labeled F-actin was added. The observed increase in fluorescence in the Cy3 and YFP-channels illustrate binding of F-actin to the SLB mediated by bright ezrinABD. A similar study was done by Janke *et al*., where full length Ezrin protein bound onto DGS-Ni-NTA but F-actin-Ezrin binding failed as C-terminus was not free due to dormant confirmation [5]. Our study employs ezrinABD domain alone thus circumventing the problem caused by full length Ezrin. Further, TIRF microscopy confirmed ezrinABD and F-Actin interaction thus validating the availability of the F-Actin binding site as seen *in-silico*. The threshold concentration of dark ezrinABD for actin binding to the SLB was 0.5 nM and actin binding saturated at higher concentrations of HEK (dark ezrinABD) (Fig 4E and 4F). Use of the combination of GST and His domain to protect delicate protein domains during purification could be a useful strategy to express a variety of proteins that would otherwise lose their activity using conventional purification strategies. The present strategy is applicable for proteins of the ERM family that allows the purification of their C-terminus alone and similar proteins containing a charged C-terminal domain.

## Conclusion

Using *in-silico* modeling of the GST tagged bright ezrinABD; we show that the GST domain which is essential for obtaining a pure protein was masked by the ezrinABD, while designed internal 10xHis tag was free. Therefore internal 10xHis was used for purification to obtain pure and functional ezrinABD. By demonstrating the ease in culminating homology modeling and imaging data to study ezrinABD and actin interaction, the efficiency and flexibility of construct is highlighted. This study proves that the strategy of using 10xHis internal tag for purification of functional 10xHis-YFP-ezrinABD at high yields was successful. This design can be used for members of ERM family proteins and other proteins as well.

## Supporting Information

S1 Fig Diagram showing arrangement of the ezrinABD protein with 10xHis, GST and MBP fusion tags. (TIF)

S2 Fig Diagram showing arrangement of engineered protein for microscopic studies. (TIF)

S3 Fig Splicing overlap PCR for YFP-ezrinABD fusion. Lane M; Marker (Gene Ruler 1kb DNA Ladder), Lane 1and 2; YFP and ezrinABD respectively, Lane 3; YFP-ezrinABD (1.08kb) fusion PCR product.
(TIF)

S4 Fig Addition of 10xHis at N-terminus of YFP-ezrinABD and KCK-ezrinABD: Agarose gel showing amplification of (a) 6xHis YFP-ezrinABD, (b) 10xHis YFP-ezrinABD, (c) 6xHis KCK-ezrinABD, (d) 10xHis KCK-ezrinABD. (TIF)

S1 Table List of primers used for construction of bright and dark ezrinABD. (DOCX)

S2 Table Plasmids used for bright and dark ezrinABD preparation. (DOCX)

S3 Table Constructs tried for expression in different strains of *E*.*coli*. (DOCX)

S1 Text Reagents. (DOCX)
