# Supplementary figures and images for: Tailor-Made Ezrin Actin Binding Domain to Probe Its Interaction with Actin In-Vitro
Source: PLoS One. 2015 Apr 10;10(4):e0123428. doi: 10.1371/journal.pone.0123428 (PMC4393143; doi:10.1371/journal.pone.0123428)

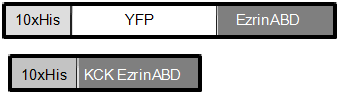

Supplement: S1 Fig — (TIF) [file pone.0123428.s001.tif]

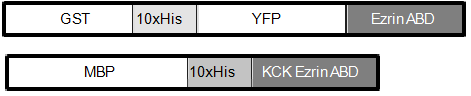

Supplement: S2 Fig — (TIF) [file pone.0123428.s002.tif]

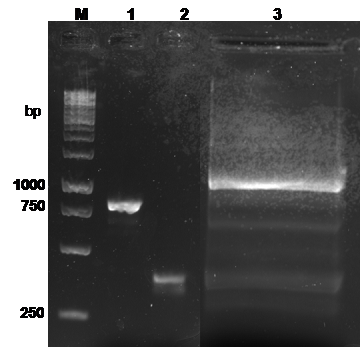

Supplement: S3 Fig — Lane M; Marker (Gene Ruler 1kb DNA Ladder), Lane 1and 2; YFP and ezrinABD respectively, Lane 3; YFP-ezrinABD (1.08kb) fusion PCR product. (TIF) [file pone.0123428.s003.tif]

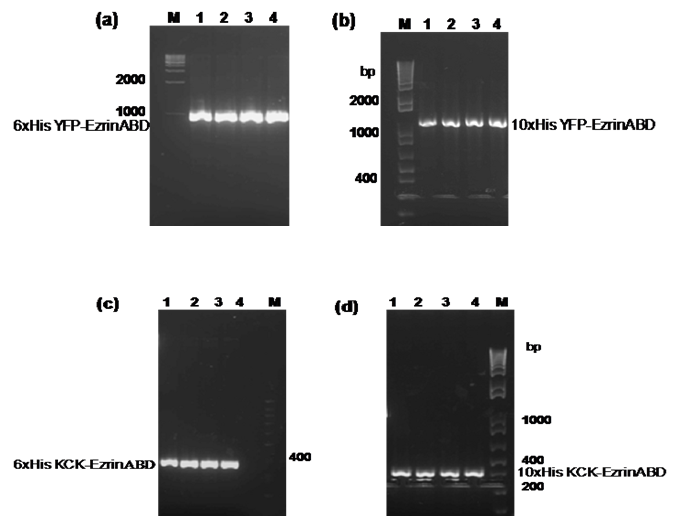

Supplement: S4 Fig — (TIF) [file pone.0123428.s004.tif]
