# Supplementary material for: Tailor-Made Ezrin Actin Binding Domain to Probe Its Interaction with Actin In-Vitro
Source: PLoS One. 2015 Apr 10;10(4):e0123428. doi: 10.1371/journal.pone.0123428 (PMC4393143; doi:10.1371/journal.pone.0123428)
Supplement: S1 Table — (DOCX) [file pone.0123428.s005.docx]

**Table S1.** List of primers used for construction of bright and dark ezrinABD

| Sr. No. | Primer Name | Restriction sites introduced by primers shown in bold | Sequence |
| --- | --- | --- | --- |
| 1 | Forward Primer YFP I(P1) | *NdeI* | 5′-GGAATTC***CATATG***GTGAGCAAGGGCGAGGAGCTG-3′ |
| 2 | Reverse Primer YFP I(P2) |  | 5′-GTAGCTCACCGGCTCGTACACCCTAGATCTGAGTCCGGACTTGTA-3′ |
| 3 | Forward Primer Ezrin I(P3) |  | 5′-TACAAGTCCGGACTCAGATCTAGGGTGTACGAGCCGGTGAGCTAC-3′ |
| 4 | Reverse Primer Ezrin I(P4) | *BamHI* | 5′-CGC***GGATCC***TTACAGGGCCTCGAACTCGTCGATG-3′ |
| 5 | Forward Primer KCK-Ezrin I(P8) | *NdeI* | 5’-GGAATTC***CATATG***AAATGCAAAGTGTACGAGCCGGTGAGCTACCATGTCC-3’ |
| 6 | Forward Primer 6xHis- YFP(P5) |  | 5’-CATCATCATCATCATCACGTGAGCAAGGGCGAGGAG-3’ |
| 7 | Forward primer 10xHis-YFPI(P6) | *BamHI* | 5’-CGCCGC***GGATCC***CATCATCATCATCATCATCATCATCATCACGTG-3’ |
| 8 | Forward Primer 6xHis- KCK(P10) |  | 5’-CATCATCATCATCATCACAAATGCAAAGTGTACGAGCCGGTG-3’ |
| 9 | Forward Primer 10xHis- KCK(P9) | *EcoRI* | 5’-CCG***GAATCC***CATCATCATCATCATCATCATCATCATCAC AAATG-3’ |
| 10 | Reverse Primer Ezrin II(P7) | *NotI* | 5’-AGGAAAAAA***GCGGCCGC***TTACAGGGCCTCGAACTCGTCGATG-3’ |
| 11 | Forward primer 10xHis-YFPII | *NdeI* | 5’-GGAATTC***CATATG***CATCATCATCATCATCATCATCATCATCACGTG-3’ |
| 12 | Forward Primer 10xHis- KCKII(P11) | *NdeI* | 5’-GGAATTC***CATATG***CATCATCATCATCATCATCATCATCATCAC AAATG -3’ |
| 13 | Reverse Primer Ezrin III(P9) | *EcoRI* | 5’-CCG***GAATTC***TTACAGGGCCTCGAACTCGTCGATG-3’ |
