# Supplementary material for: Tailor-Made Ezrin Actin Binding Domain to Probe Its Interaction with Actin In-Vitro
Source: PLoS One. 2015 Apr 10;10(4):e0123428. doi: 10.1371/journal.pone.0123428 (PMC4393143; doi:10.1371/journal.pone.0123428)
Supplement: S2 Table — (DOCX) [file pone.0123428.s006.docx]

**Table S2.** Plasmids used for bright and dark ezrinABD preparation

| **Plasmids** | **Specification** | **Reference** |
| --- | --- | --- |
| pET-15b YFP-ezrinABD | Splicing overlap PCR amplified 1.068kb YFP-ezrinABD was cloned in *NdeI* and *BamHI* sites of pet15b vector | This study |
| pET-15b KCK-ezrinABD | PCR amplified 339bp KCK-ezrinABD was cloned in *NdeI* and *BamHI* sites of pet15b vector | This study |
| pGEX-4T-1 10xHis YFP-ezrinABD | 10xHis was added to YFP-ezrinABD by PCR and 1.098kb cloned in pGEX 4T-1 vector at *BamHI* and *NotI* sites. | This study |
| pGEX-4T-1 10xHis KCK-ezrinABD | 10xHis was added to KCK-ezrinABD by PCR and 369bp cloned in pGEX 4T-1 vector at *EcoRI* and *NotI* sites. | This study |
| pMAL-c5X 10xHis YFP-ezrinABD | 10xHis was added to YFP-ezrinABD by PCR and 1.098kb cloned in pMALc5X vector at *NdeI* and *EcoRI* sites. | This study |
| pMAL-c5X 10xHis KCK-ezrinABD | 10xHis was added to KCK-ezrinABD by PCR and 369bp cloned in pGEX 4T-1 vector at *NdeI* and *EcoRI* sites. | This study |
