# Supplementary material for: Tailor-Made Ezrin Actin Binding Domain to Probe Its Interaction with Actin In-Vitro
Source: PLoS One. 2015 Apr 10;10(4):e0123428. doi: 10.1371/journal.pone.0123428 (PMC4393143; doi:10.1371/journal.pone.0123428)
Supplement: S3 Table — (DOCX) [file pone.0123428.s007.docx]

**Table S3.** Constructs tried for expression in different strains of *E.coli*.

| ExpressionVector and insert name | Expression Strain | IPTG concentration | Temperature |
| --- | --- | --- | --- |
| pET-15b(YFP-ezrinABD), pET-15b(KCK-ezrinABD) | BL21(DE3), BL21(DE3)star, BL21(DE3) plysS | 0.2, 0.4, 0.6, 0.8 and 1mM | 20°, 25° and 30°C |
| pET-15b(YFP-ezrinABD), pET-15b(KCK-ezrinABD) | C41(DE3), RIL codon plus | 0.2, 0.4, 0.6, 0.8 and 1mM | 20°, 25° and 30°C |
| pGEX-4T-1(10xHis-YFP-ezrinABD) | BL21(DE3) | 0.3mM, 0.4mM | 18°C, 30°C |
| pMAL-c5X(10xHis-YFP-ezrinABD) | BL21(DE3) star | 0.4mM | 30°C |
| pGEX-4T-1(10xHis-KCK-ezrinABD) | BL21(DE3) | 0.1mM, 0.4mM | 18°C ,30°C |
| pMAL-c5X(10xHis-KCK-ezrinABD) | BL21(DE3) star | 0.4mM | 30°C |
