# Supplementary material for: Tailor-Made Ezrin Actin Binding Domain to Probe Its Interaction with Actin In-Vitro
Source: PLoS One. 2015 Apr 10;10(4):e0123428. doi: 10.1371/journal.pone.0123428 (PMC4393143; doi:10.1371/journal.pone.0123428)
Supplement: S1 Text — (DOCX) [file pone.0123428.s008.docx]

**Reagents:**

Lipids (Avanti Polar Lipids, USA), Hellmanex III special cleaning concentrate (Hellma Analytics, Germany), pMAL c5X plasmid, Restriction enzymes, Polymerase, dNTP’s, Thermopol Buffer and Quick Ligase were purchased from New England Biolabs, His Trap HP prepacked column, Glutathione Beads and prepacked columns, Superdex 75 10/300 GL were purchase from GE, USA. All other reagents were procured from Sigma unless otherwise mentioned. G-Actin was obtained from chicken skeletal muscle after a protocol from J. Spudich's group (Pardee and Spudich, 1982) and partially labeled with malemeide-Cy3 (Lumiprobe, Florida, USA) using standard protocols.
